# A nonsense mutation in the *beta-carotene oxygenase 2 (BCO2) *gene is tightly associated with accumulation of carotenoids in adipose tissue in sheep (*Ovis aries*)

**DOI:** 10.1186/1471-2156-11-10

**Published:** 2010-02-02

**Authors:** Dag I Våge, Inger A Boman

**Affiliations:** 1 Centre for Integrative Genetics (CIGENE), Dept of Animal and Aquacultural Sciences, Norwegian University of Life Sciences (UMB), PO Box 5003, N-1432 Ås, Norway; 2 The Norwegian Association of Sheep and Goat Breeders, PO Box 104, N-1431 Ås, Norway

## Abstract

**Background:**

Sheep carcasses with yellow fat are sporadically observed at Norwegian slaughter houses. This phenomenon is known to be inherited as a recessive trait, and is caused by accumulation of carotenoids in adipose tissue. Two enzymes are known to be important in carotenoid degradation in mammals, and are therefore potential candidate genes for this trait. These are *beta-carotene 15,15'-monooxygenase 1 (BCMO1) *and the *beta-carotene oxygenase 2 (BCO2)*.

**Results:**

In the present study the coding region of the *BCMO1 *and the *BCO2 *gene were sequenced in yellow fat individuals and compared to the corresponding sequences from control animals with white fat. In the yellow fat individuals a nonsense mutation was found in *BCO2 *nucleotide position 196 (*c.196C>T*), introducing a stop codon in amino acid position 66. The full length protein consists of 575 amino acids. In spite of a very low frequency of this mutation in the Norwegian AI-ram population, 16 out of 18 yellow fat lambs were found to be homozygous for this mutation.

**Conclusion:**

In the present study a nonsense mutation (*c.196C>T*) in the *beta-carotene oxygenase 2 (BCO2) *gene is found to strongly associate with the yellow fat phenotype in sheep. The existence of individuals lacking this mutation, but still demonstrating yellow fat, suggests that additional mutations may cause a similar phenotype in this population. The results demonstrate a quantitatively important role for BCO2 in carotenoid degradation, which might indicate a broad enzyme specificity for carotenoids. Animals homozygous for the mutation are not reported to suffer from any negative health or development traits, pointing towards a minor role of BCO2 in vitamin A formation. Genotyping AI rams for *c.196C>T *can now be actively used in selection against the yellow fat trait.

## Background

The sporadic occurrence of yellow fat in sheep is known to be caused by accumulation of carotenoids in adipose tissue, mainly xanthophylls derived from their plant containing diet [1-3]. Animals are unable to synthesise vitamin A *de novo*, and are therefore dependent on metabolising β-carotene, and other carotenoids, into vitamin A. The central step in formation of vitamin A is the enzymatic cleavage of β-carotene. Two key enzymes responsible for this have been characterised at the molecular level in multiple organisms, including mammals. These are the *beta-carotene 15,15'-monooxygenase 1 *(*BCMO1*) responsible for the symmetric cleavage of β-carotene into two molecules of retinal [4-6], and the *beta-carotene oxygenase 2 *(*BCO2*) responsible for the asymmetric cleavage of β-carotene into β-apo-10'-carotenal (C_27_) and β-ionone (C_13_) [7].

Some recent studies have confirmed that naturally occurring mutations in *BCMO1 *and *BCO2 *have significant impact on the carotenoid metabolism in diverse organisms. In domestic chicken, yellow skin was found to be caused by a regulatory mutation that inhibits expression of the *BCO2 *gene in skin, allowing deposition of yellow carotenoids [8]. In cows, the introduction of a premature stop codon in the *BCO2 *gene caused an increased level of β-caroten in milk and serum [9]. In humans, four SNPs in or close to the *BCMO1 *gene showed a robust association to β-carotene plasma levels [10].

Several reports have concluded that 'yellow fat' in lamb carcasses is inherited as a simple recessive trait [11,12]. A deficiency in the 'xanthophyll oxidase' system was early suggested as a probable explanation of this trait in sheep [1]. Based upon this, and the above mentioned studies of genetic variation in the *BCMO1/BCO2 *genes, we hypothesised that a mutation in one of these genes could explain the recessively inherited yellow fat observed in Norwegian sheep. In the present study we have sequenced the coding region of both *BCMO1 *and *BCO2 *in yellow fat sheep and white fat control animals.

## Results

Sequencing of the BCMO1 coding region in the two ewes giving yellow fat offspring, together with the control ewe, did not reveal any genetic differences that were likely to explain the yellow fat phenotype (GenBank accession no. FN543098).

**Figure 1:**
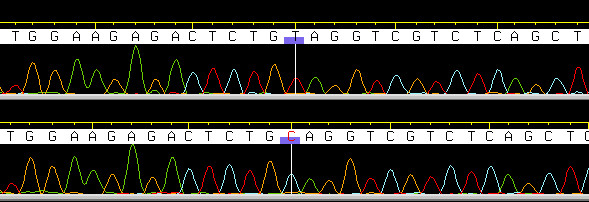
**The nonsense ovine *BCO2 *mutation**. The figure is showing the sequence chromatograms from an individual homozygous for the *c.196C>T *mutation (upper line) and one control individual without the mutation (lower line). The sequences were assembled and viewed with Phred/Phrap/Consed software.

**Figure 2:**
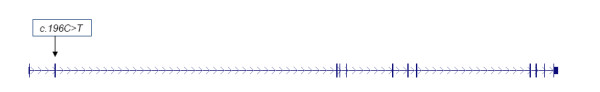
**The *BCO2 *gene structure**. The figure is showing the predicted gene structure of the ovine BCO2 gene, based on the bovine genomic sequence. The nonsense *c.196C>T *mutation is localized in the 2nd exon, assumed to produce a truncated protein of 65 amino acid residues (CAX63048), compared to 575 amino acid residues in the wild type version (CAX63047). The figure is modified from the UCSC Genome Browser.

Comparing the coding part of the *BCO2 *cDNA sequence in 2 white fat control lambs (GenBank accession no. FN257486) and 4 yellow fat (GenBank accession no. FN257487) lambs collected from the abattoir, a substitution of cytosine with thymine (Figure 1) was identified in nucleotide position 196 (nt-numbering according to translation start codon) in 3 out of 4 yellow fat lambs. This substitution (*c.196C>T*) introduced a stop codon in amino acid position 66. Based on the bovine gene structure, the location of the nonsense mutation is predicted to be in the second exon of the *BCO2 *gene (Figure 2). Additional non-synonymous substitutions were observed downstream of the *c.196C>T *(Table 1), but we assume that this part of the protein is not translated in the animals homozygous for *c.196C>T*. The yellow fat lamb without the *c.196C>T *mutation carried a *BCO2 *haplotype that is more similar to the haplotypes found in the white fat control lambs, when comparing the additional 5 variable positions that were sequenced (Table 1). In addition to *c.196C>T, c.478C>T *and *c.943T>A *were verified by genotyping in a larger number of animals, and submitted to dbSNP (NCBI_ss# BCO2-196 181341842, BCO2-478 181341843, BCO2-943 181341844).

**Table 1:** Polymorphic positions in the *BCO2 *gene

		***196C>T***	***478C>T***	***943T>A***	***1265A>T***	***1292C>G***	***1310T>C***
ID	Phenotype	Q66X	R160C	F315I	N422I	A431G	V437A
5002	Yellow	XX	*CC*	*II*	*NN*	*GG*	*AA*
5004	Yellow	XX	*CC*	*II*	*NN*	*GG*	*AA*
5013	Yellow	QQ	RC	FF	NI	AA	VV
5023	Yellow	XX	*CC*	*II*	*NN*	*GG*	*AA*
5028	White	QQ	RC	FI	NN	AA	VV
5080	White	QQ	RR	FI	NN	AA	VV

The six polymorphic sites influencing the amino acid sequence of *BCO2 *in 4 yellow fat individuals and 2 controls. The nucleotide positions are numbered according to first base of the translation start codon of the ovine *BCO2 *gene (FN257486). *c.196C>T, c.478C>T *and *c.943T>A *are verified by additional genotyping and submitted to dbSNP (NCBI_ss# BCO2-196 181341842, BCO2-478 181341843, BCO2-943 181341844). The amino acids shown in italic are assumed to not be translated.

In addition to the 6 sequenced individuals, 14 yellow fat lambs were genotyped for *c.196C>T*. The results showed that 13 individual were homozygous for *c.196C>T*, while the last animal did not have the mutation. The two ewes giving yellow fat lambs were both heterozygous for *c.196C>T*. Genotyping of 411 AI rams born in the period 1977-2006 revealed that 7 were heterozygous and 2 homozygous for *c.196C>T*. Only 3 of the 9 rams with one or two copies of the *c.196C>T *mutation were previously revealed to have yellow fat allele(s) based on progeny testing. Also, 7 AI rams progeny tested to have yellow fat allele(s), did not have *c.196C>T*. The most recent Norwegian White Sheep (NWS) ram carrying the mutation was born in 1997. A summary of the genotyping results are given in Table 2. The frequency of individuals with one or two copies of *c.196C *> T in the yellow fat group was found to be significantly different from the frequency in the white fat group (P = 7.44 × 10^-21^), calculated by Fisher exact test as described in Methods.

**Table 2:** Distribution of *BCO2 c.196 *genotypes in different phenotypic groups

	*c.196 *genotype		
Phenotype	CC	CT	TT
Lambs with yellow fat	1		13
Ewes giving yellow fat offspring		2	
White fat control animals	1		
AI rams progeny tested to have yellow fat allele(s)	7	2	1
AI rams progeny tested to not have yellow fat allele(s)	395	5	1

All animals, except the 4 yellow fat lambs and the 2 control lambs initially sequenced to identify the BCO2 *c.196C>T *mutation, are included in this table.

## Discussion

The *BCO2 *mutation (*c.196C>T*) identified in the present study showed a highly significant (P = 7. 44 × 10^-21^) association with yellow adipose tissue in 428 genotyped sheep. In spite of the low allele frequency of this mutation among the 411 NWS AI rams (0.0134), 16 out of 18 yellow fat individuals were homozygous for this mutation, and both ewes with yellow fat offspring were heterozygous. The low frequency of the *c.196C>T *allele among the AI rams indicates a low frequency also in the NWS ewe population. An AI ram must be mated to a ewe with one or two copies of the *c.196C>T *allele to possibly be revealed as a carrier by progeny testing. If we assume a similar allele frequency among the ewes, less than 3% of the ewes will carry the *c.196C>T *allele. This may explain why only 3 out of 9 AI rams with one or two copies of the *c.196C>T *allele were revealed to have yellow fat allele(s) by progeny testing.

While the mutant allele encodes a truncated protein of only 65 amino acid residues, the *BCO2 *protein length in the control animals is 575 amino acids. It can therefore be hypothesised that the mutant *BCO2 *version generates a non-functional enzyme. However, we cannot completely exclude the possible existence of functional splice variants of *BCO2*. In that case, a closely linked mutation outside the *BCO2 *coding region could be the causal one. However, splicing out the second exon, harbouring the *c.196C>T *nonsense mutation, will reduce the protein size by at least 68 amino acids (positions 30-97), assuming an intron/exon structure similar to the bovine *BCO2 *gene (Figure 2) [13]. Such a large rearrangement will most likely impair enzyme function, and we consider this as a less likely scenario, given that the generation of a truncated protein of 65 amino acids can fully explain the observed phenotype.

Based on our findings, and the fact that *BCO2 *mutations in chicken and cattle have resulted in accumulation of carotenoids in skin and milk/serum respectively [8,9], we suggest that this mutation is causing the yellow fat phenotype in the majority of the NWS individuals observed. A loss of function mutation like this is also in agreement with the recessive inheritance of yellow fat in sheep reported earlier [11,12].

Interestingly, two lambs found to have the yellow fat phenotype did not display this genetic mutation. One of these lambs was sequenced (5013), and it was noticed that the haplotype of the 6 polymorphic amino acid positions of this lamb differed from the controls only in being homozygous FF at amino acid position 315 and heterozygous NI at amino acid position 422 (Table 1). Whether one of these differences, or additional mutations outside the *BCO2 *coding region, is causing the yellow fat phenotype is not possible to determine based on these two individuals. The fact that 7 AI rams were progeny tested to have yellow fat allele(s) without having the *c.196C>T *mutation, may also point towards a possible existence of additional mutations causing yellow fat in this population. However, we cannot completely exclude the possibility of erroneous classification of an AI ram with regard to yellow fat status, caused by wrong parentage assignment of a yellow fat litter.

**Figure 3:**
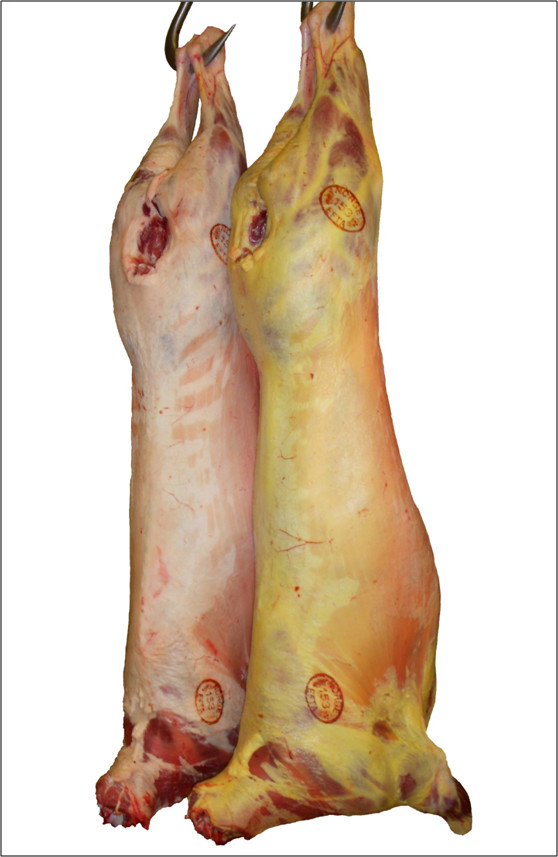
**The yellow fat phenotype in sheep**. The picture is showing a typical yellow fat carcass in front of a normal coloured carcass.

In an earlier paper the amount of carotenoids in perirenal fat in three typical Norwegian 'yellow fat' animals was estimated to be 220 μg/100 g fat [14]. In the same study, the concentration of total carotenoid in perirenal fat was found to vary between 12 and 236 μg/100 g fat, with a good correlation (r = 0.85) between visual score and measured amounts of total carotenoid. Based on the visual appearance (Figure 3), the amount of carotenoids deposited in the adipose tissue in yellow fat individuals is considerably elevated compared to the control animals, indicating a significant role of the BCO2 enzyme in carotenoid degradation in quantitative terms. This is also in agreement with the observation that cows homozygous for a similar deleterious *BCO2 *mutation produce milk with 78% more β-carotene compared to cows without the mutation [9].

The main carotenoids in adipose tissue from yellow fat sheep are lutein and flavoxanthin, while only small amounts of β-carotene are found [1,2]. A study of cleavage activity for recombinant *BCMO1 *indicate that β-carotene is much more efficiently cleaved compared to other carotenoids [6]. The present results may indicate that the BCO2 enzyme is a less selective carotenoid cleaver. If so, this would explain why the BCO2 function seems to be quantitatively important for carotenoid degradation in tissues with a high non-β-carotene content, in spite of an apparently functional *BCMO1 *gene.

The relative importance of symmetric versus asymmetric cleavage of β-carotene in vitamin A formation has been debated for a long time. Recent knockout studies in zebrafish and mice have strongly indicated that *BCMO1 *is the key enzyme in production of vitamin A from β-carotene [15,16]. A similar study in mice also indicated that *BCMO1 *plays a major role in conversion of β-carotene to retinoids, but the authors did not exclude a possible contribution of other enzymes like e.g. eccentric cleavage enzymes [17]. It was recently shown that a deleterious mutation in the bovine *BCO2 *gene caused an increased level of β-carotene in milk and serum, and a reduced level of vitamin A in liver [9]. However, neither the cows nor the sheep seem to encounter any adverse developmental or health effects as a result of having an impaired BCO2 function. This does not exclude the possibility that BCO2 might play a role in vitamin A formation in these ruminants, but its contribution seems not to be indispensable.

Although the presence of increased amounts of carotenoids in adipose tissue is not known to negatively affect the sheep, or the sheep meat consumers, it constitutes a marketing problem due to the visual appearance. In the Norwegian sheep breeding scheme this has until now been handled by culling sires giving yellow fat offspring. According to Animalia - Meat and Poultry Research Centre, the frequency of yellow fat lamb carcasses in Norway was only 0.02% in 2008. This implies it is hard to identify individuals carrying heritable yellow fat, simply because the probability of mating two carriers is very low. Selection against this trait has now been made more efficient by genotyping all potential AI-rams for the presence of the *c.196C>T *mutation.

## Conclusions

The majority of yellow fat individuals are found to be homozygous for the low frequency *BCO2 c.196C>T *mutation, which most likely results in a truncated and functionally impaired protein. We therefore suggest that this mutation is a cause of yellow fat in sheep. However, two lambs with yellow fat and 7 AI rams progeny tested to have yellow fat allele(s) did not have this mutation, suggesting that additional mutations might exist in this population. The large difference in carotenoid content between yellow and white fat carcasses show that BCO2 mediated degradation of carotenoids is quantitatively important. The reason might be that BCO2 have a broader specificity for carotenoids other than β-carotene, compared to BCMO1. No adverse health or development effects are reported on yellow fat animals, suggesting a non-essential BCO2 contribution in vitamin A formation. Culling AI rams carrying *c.196C>T *will reduce the problem with yellow fat in populations having this mutation.

## Methods

### Animals

Liver samples of approximately 0.5 × 0.5 cm was collected in RNA*later*™ (QIAGEN, Hilden, Germany) from 2 ewes that both have given birth to yellow fat lambs, together with a randomly selected control animal (typical phenotypes are shown in Figure 3). Additional liver samples were taken from 18 yellow fat lambs carcasses in two different Norwegian slaughter houses, together with two control animals from clearly white fat carcasses. In addition, biological samples for DNA isolation were collected from 411 Norwegian White Sheep (NWS) AI rams born in the period 1977-2006.

### RNA isolation and cDNA synthesis

Total RNA was extracted with TRIzol^® ^(Invitrogen, Carlsbad, CA, USA). The isolated RNA was treated with DNase I (Applied Biosystems). Synthesis of cDNA was performed using a poly dT-primer and SuperScript™ II Reverse Transcriptase (Invitrogen). RNA (1 ug) was added to each reaction, in a reaction volume totalling 20 ul.

### PCR amplification, cloning and sequencing of *BCMO1*

The ovine *BCMO1 *coding region was amplified by primer pairs F0025/R0673, F0066/R1464, F0157/R1464 and F1226/R1976 (Table 3). The primers were designed based on an alignment of the bovine *BCMO1 *sequence (NM_001024559.1) and the human *BCMO1 *sequence (NM_017429.1). DNA was initially denatured for 10 min at 95°C and thereafter amplified for 40 cycles at 95°C for 30 sec, 57°C for 30 sec and 72°C for 1.5 min using AmpliTaq Gold^® ^(Applied Biosystems, Foster City, CA, USA). The resulting fragments were cloned into the pGEM^®^-T Easy Vector (Promega, Madison, WI, USA) and sequenced. Plasmids were sequenced with primers Sp6 and M13, and the BigDye^® ^Terminator v3.1 kit (Applied Biosystems) was used.

**Table 3:** Primers used for amplification and sequencing the ovine *BCMO1 *and *BCO2 *genes

Name	Direction	Position	Sequence5'-3'
*BCMO1 *- primers			
F0025	Forward	-109 to -90^a^	CATCTGAAGGGAGGGAGATG
F0066	Forward	-85 to -66^b^	AAGACAAGGAGTGGCCAAGA
F0157	Forward	46-65^b^	GTGAGGGCCARAGTRACAGG
R0673	Reverse	520-539^b^	TTTCCAGCAGCATCGTAGTG
F1226	Forward	1092-1111^b^	CGTGGACAAGAATGCAGAAG
R1464	Reverse	1334-1352^b^	CTCCTCTCTCCACGTCAAGG
R1976	Reverse	1826-1845^a^	GATAGTCCTCACGGCCAAAA
*BCO2 *- primers			
7541	Forward	-25 to -6^c^	CTGCTGCTGCAGAACTCAAC
7542	Reverse	648-666^d^	ATGTGCAGTTGCTCCGTTC
7543	Forward	591-611^d^	GGACATTGAAACCCTGGAAAA
7544	Reverse	1392-1404^d^	CCATTGAATTGGCCATAGTTG
7545	Forward	1287-1306^d^	TTCAGCCAGTGCTGTGAAAC
7546	Reverse	1758-1777^c^	AGCGAGCACATTCATTCAAA
7547	Forward	-52 to -34^c^	AGCGTGAGGATTTGGGAAT
7548	Reverse	588-607^d^	CCAGGGTTTCAATGTCCACT
7550	Forward	104-126^d^	TGGAACAGACTCATCAGAAAACA
7551	Reverse	257-276^d^	GAATTTCCCAGGTCCAACAC
BCO2F	Forward	170-189^d^	TTCTAACCACGGTGGAAGAG
BCO2R	Reverse	230-249^d^	ATAGCCATTGAGCCACTCAG
BCO2E	Forward	177-195^d^	CACGGTGGAAGAGACTCTG

a. Positions are numbered according to first base of the translation start codon of the bovine *BCMO1 *gene (NM_001024559.1).

b. Positions are numbered according to first base of the translation start codon of the ovine *BCMO1 *gene (FN543098)

c. Positions are numbered according to first base of the translation start codon of the bovine *BCO2 *gene (NM_001101987).

d. Positions are numbered according to first base of the translation start codon of the ovine *BCO2 *gene (FN257486).

### PCR amplification and sequencing of *BCO2*

Primers 7541-7548 were designed based on the bovine *BCO2*-sequence available in GenBank (NM_001101987). Primers 7550 and 7551 were designed based on ovine *BCO2*-sequence (FN257486).

0.5 ul of the cDNA reaction mix was used as template for PCR amplification of the coding region of *BCO2 *with the following primer combinations; 7541-7542, 7543-7544, 7545-7546, 7547-7548. DNA was denatured for 10 min at 95°C and PCR run for 40 cycles at 95°C for 30 sec, 57°C for 30 sec and 72°C for 1.5 min using AmpliTaq Gold^® ^(Applied Biosystems). The resulting fragments were directly sequenced with primers 7541, 7542, 7543, 7544, 7545, 7546, 7547 using the BigDye^® ^Terminator v3.1 kit (Applied Biosystems). cDNA from 4 yellow fat individuals and 2 control animals were amplified and sequenced.

### A PCR-RFLP method for detecting the *BCO2 *nonsense mutation

A PCR-RFLP was developed to detect the nonsense mutation found in the sheep *BCO2 *gene. Primers 7550 and 7551 were used to amplify a 173 bp region containing the *c.196C>T *mutation localized to amino acid position 66. PCR was carried out in a 20 ul reaction containing 10 pmol of each primer and 20 ng DNA, and was run for 35 cycles at 95°C for 15 s, 60°C for 15 s, and 72°C for 15 s. The PCR fragment was cut with HpyCH4V at 37°C for 2 hours. If the wild type allele is present, the reaction will produce fragments of 81, 55, 32 and 5 bp. If the nonsense allele is present, HpyCH4V will produce fragments of 113, 55 and 5 bp.

An assay for the MassARRAY platform (SEQUENOM, San Diego, USA) was developed for high throughput genotyping. The region surrounding *c.196C>T *was amplified with primers BCO2F and BCO2R, while the primer extension reaction was carried out with the primer BCO2E. All animals included in the study were genotyped by this assay, including those initially tested by the PCR-RFLP assay.

### Statistical testing

To make an independent test on the association between the *c.196C>T *allele and the yellow fat phenotype, a 2 × 2 contingency table was established, including all individuals (434) except the 6 lambs initially sequenced for identifying the mutation. Lams showing the yellow fat phenotype at slaughter, and individuals with yellow fat offspring, were included in the yellow fat phenotypic group (26). The remaining animals (402) were in the white fat phenotypic group. Individuals homozygous or heterozygous for *c.196C>T *(24) constituted one genotypic group, the other genotypic group constituted animals being homozygous CC at cDNA position 196 (404). The individuals included in the test are shown in Table 2.

## Competing interests

The authors declare that they have no competing interests.

## Authors' contributions

IAB conceived the study together with DIV and coordinated the sample collection. IAB also analysed the genotyping data. DIV was responsible for the molecular genetics work, analysed the DNA sequence data, and wrote the manuscript together with IAB. Both authors read and approved the final manuscript.
